# Computational pathology applied to clinical colorectal cancer cohorts identifies immune and endothelial cell spatial patterns predictive of outcome

**DOI:** 10.1002/path.6378

**Published:** 2025-01-09

**Authors:** Nicholas Trahearn, Chirine Sakr, Abhirup Banerjee, Seung Hyun Lee, Ann‐Marie Baker, Hemant M Kocher, Valentina Angerilli, Federica Morano, Francesca Bergamo, Giulia Maddalena, Rossana Intini, Chiara Cremolini, Giulio Caravagna, Trevor Graham, Filippo Pietrantonio, Sara Lonardi, Matteo Fassan, Andrea Sottoriva

**Affiliations:** ^1^ Centre for Evolution and Cancer The Institute of Cancer Research London UK; ^2^ UCL Cancer Institute UCL London, UK; ^3^ Barts Cancer Institute Queen Mary University of London London UK; ^4^ Systems Oncology group, Cancer Research UK Manchester Institute The University of Manchester Manchester UK; ^5^ Department of Medicine (DIMED) University of Padua Padua Italy; ^6^ Fondazione IRCCS Istituto Nazionale dei Tumori Milan Italy; ^7^ Veneto Institute of Oncology IOV‐IRCCS Padua Italy; ^8^ Department of Surgical, Oncological and Gastroenterological Sciences University of Padua Padua Italy; ^9^ Computational Biology Research Centre Human Technopole Milan Italy; ^10^ University of Triest Triest Italy

**Keywords:** computational pathology; artificial intelligence; colorectal cancer; endothelial; lymphocyte; macrophage

## Abstract

Colorectal cancer (CRC) is a histologically heterogeneous disease with variable clinical outcome. The role the tumour microenvironment (TME) plays in determining tumour progression is complex and not fully understood. To improve our understanding, it is critical that the TME is studied systematically within clinically annotated patient cohorts with long‐term follow‐up. Here we studied the TME in three clinical cohorts of metastatic CRC with diverse molecular subtype and treatment history. The MISSONI cohort included cases with microsatellite instability that received immunotherapy (*n* = 59, 24 months median follow‐up). The BRAF cohort included BRAF V600E mutant microsatellite stable (MSS) cancers (*n* = 141, 24 months median follow‐up). The VALENTINO cohort included RAS/RAF WT MSS cases who received chemotherapy and anti‐EGFR therapy (*n* = 175, 32 months median follow‐up). Using a Deep learning cell classifier, trained upon >38,000 pathologist annotations, to detect eight cell types within H&E‐stained sections of CRC, we quantified the spatial tissue organisation and colocalisation of cell types across these cohorts. We found that the ratio of infiltrating endothelial cells to cancer cells, a possible marker of vascular invasion, was an independent predictor of progression‐free survival (PFS) in the BRAF+MISSONI cohort (*p* = 0.033, HR = 1.44, CI = 1.029–2.01). In the VALENTINO cohort, this pattern was also an independent PFS predictor in *TP53* mutant patients (*p* = 0.009, HR = 0.59, CI = 0.40–0.88). Tumour‐infiltrating lymphocytes were an independent predictor of PFS in BRAF+MISSONI (*p* = 0.016, HR = 0.36, CI = 0.153–0.83). Elevated tumour‐infiltrating macrophages were predictive of improved PFS in the MISSONI cohort (*p* = 0.031). We validated our cell classification using highly multiplexed immunofluorescence for 17 markers applied to the same sections that were analysed by the classifier (*n* = 26 cases). These findings uncovered important microenvironmental factors that underpin treatment response across and within CRC molecular subtypes, while providing an atlas of the distribution of 180 million cells in 375 clinically annotated CRC patients. © 2025 The Author(s). *The Journal of Pathology* published by John Wiley & Sons Ltd on behalf of The Pathological Society of Great Britain and Ireland.

## Introduction

Colorectal cancer (CRC) is a disease with varied histological presentation and heterogeneous clinical outcome. In particular, microsatellite stable (MSS) and microsatellite instable (MSI) CRC subtypes are known to have substantial differences in terms of treatment response [1, 2, 3] and likelihood of disease progression. Immune checkpoint inhibition‐based therapies have produced notable improvements in outcome for patients with MSI cancers, but this has yet to be reflected in MSS cancers. This successful treatment pathway highlights the important role that the immune microenvironment plays within CRC, but also raises questions about its behaviour in MSS tumours. There are also other key parameters that are of importance in CRC, such as pathological subtypes (e.g. mucinous adenocarcinoma and signet ring cell carcinoma) and presence of lymphovascular invasion. However, further insight is needed to understand how the interaction between the tumour and its microenvironment influences disease progression, and thus explain the heterogeneous responses to treatment we observe within CRC.

By studying the cellular composition of tissue in and around a tumour, we can quantify aspects of the microenvironment that may be contributing to poorer outcomes. Tumour‐associated immune cells, for instance, are of particular relevance in the context of CRC, with significant attention being given to the influence of tumour‐infiltrating lymphocytes (TILs) within CRCs [4, 5, 6] and their impact on patient outcomes. In previous studies, substantial differences were observed in the numbers of TILs in immune cold MSS and immune hot MSI‐H cancers [7, 8, 9]. Macrophages, by contrast, have been shown to exhibit diverse behaviour in CRC patients. Studies showed both positive and negative outcomes for the presence of tumour‐associated macrophages [10, 11, 12, 13]. Additionally, the contrast between the effectiveness of immunotherapies in MSI‐H tumours and the very poor outcomes seen in trials on patients with MSS tumours raises questions regarding how the efficacy of immunotherapy may be associated with the biological state of these tumours at the time of diagnosis.

To gain a more comprehensive understanding of how a tumour interacts with its stromal and immune cellular compartments and how this relates to patient outcomes, a quantitative assessment of the tumour microenvironment (TME) is necessary. However, systematic evaluations of this kind are challenging to perform on a large scale because they normally require extensive manual annotation and validation by an experienced pathologist. In addition, manual histological review has several limitations, including intra‐ and inter‐observer variability, lack of precise quantification, and constraints on the proportion of each sample that can be analysed [14, 15, 16].

High‐throughput digital slide scanners and novel image analysis tools have enabled the rapid growth of the field of computational pathology, which offers an alternative to the traditional approach of manual visual assessment. In this computational paradigm, biological structures, such as individual cells, can be automatically extracted from a digitised image of a slide and used to compute complex tissue‐level metrics [17], thereby providing a consistent and quantitative assessments of cancer and tissue microenvironment, at a scale beyond what an experienced pathologist can realistically provide. This automated approach lends itself directly to a data‐driven study of the TME. Moreover, computational histopathology is already beginning to change the way diagnostic cancer specimens are assessed, paving the way for the integration of new tools based on artificial intelligence (AI) [18, 19, 20] and the identification of novel clinical biomarkers [21, 22, 23, 24, 25].

**Figure 1:**
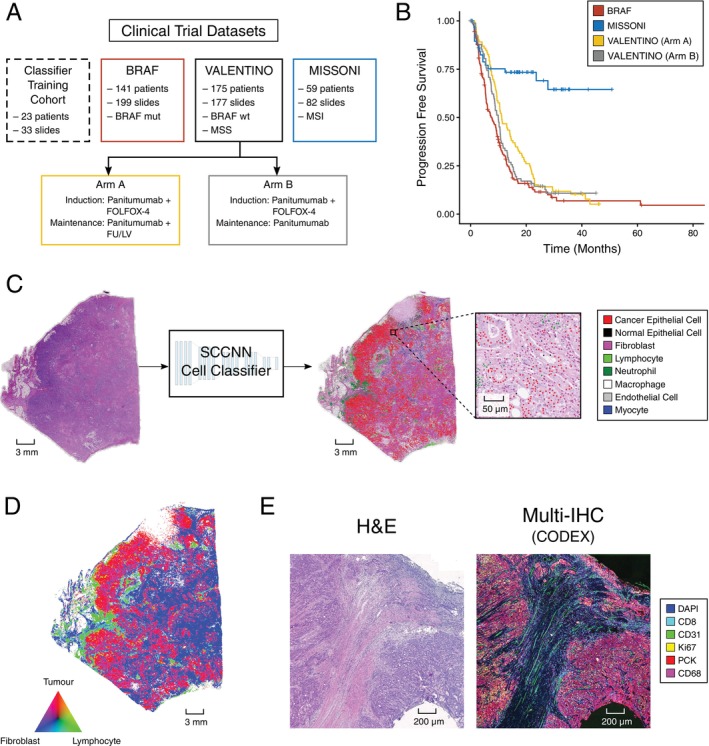
Study design. (A) Summary of CRC datasets used in classifier training and cell colocalisation analysis. (B) PFS of analysed cohorts. VALENTINO patients are divided by trial arm. (C) Example of DenseNet‐based DL model input and output on a WSI from the MISSONI cohort. Cell classifier is capable of identifying eight different cell types. (D) Cell density map of WSI shown in (C), demonstrating a per‐region‐level view of cellular balance within the section. (E) Matched and coregistered H&E and CODEX images of a CRC section from BRAF cohort. Images are of the same section, so individual cells can be matched directly between both modalities following coregistration.

For computational pathology to have clinical impact, it is critical that these methods be applied in well‐defined and clinically annotated patient cohorts with long‐term follow‐up information (>24 months), in line with previous studies [18, 26, 27]. In this study, we analysed 458 pre‐treatment sections from 375 patients from three CRC cohorts (Figure 1A, and supplementary material, Table S1). The three cohorts encompass three major molecular subtypes of CRC patients. The MISSONI cohort [28] comprises metastatic colorectal carcinomas (mCRCs) with deficient mismatch repair (dMMR)/MSI‐high (MSI‐H) that received immunotherapy. The BRAF cohort comprises mostly right‐sided *BRAF* V600E mutant mCRCs. Finally, the VALENTINO cohort [29] consists of a trial subgroup of patients with MSS, RAS/BRAF WT mCRC. All three cohorts have extensive clinical annotation, with >24 months of follow‐up information. Progression‐free survival (PFS) is different between the cohorts, with dMMR/MSI‐H CRCs having a very favourable prognosis mainly due to the efficacy of immunotherapy and *BRAF* V600E mutant MSS cancers having a poor prognosis (Figure 1B).

To investigate the TME of these varied CRC cohorts at a tissue level and shine a light upon some of the dynamics underpinning response to treatment, we opted to quantify and compare the cellular composition of the TME across these samples. Thus, using manual annotations from an experienced gastrointestinal pathologist, we trained a single‐cell deep learning (DL) classifier capable of identifying eight cell types (Figure 1C). Using the detected cells, we developed metrics of tumour invasion, which we used to identify spatial patterns of interaction between cancer and stromal cells, revealing independent predictors of survival across cohorts. We validated our cell classifier using highly multiplexed immunofluorescence (mIF) on 26 cases from the BRAF and MISSONI cohorts.

## Materials and methods

### Ethical approval and patient consent

Ethical approval was given by local ethics committees (BRAF: Oncologic Institute of Veneto, 2017/34. Missoni: Fondazione IRCCS Istituto Nazionale dei Tumori di Milano Institutional Review Board, INT 117/15. Valentino: Fondazione IRCCS Istituto Nazionale dei Tumori di Milano Institutional Review Board, INT 70/15, EPICC: Cancer Tissue Bank, 2015/2/QM/TG/CaCOL). Consent was received from all patients involved in the study. All samples were handled in compliance with the Declaration of Helsinki. Clinical and sample data were managed using anonymous numerical codes. Research was performed in accordance with local and national ethics standards.

### Data acquisition and pre‐processing

Digital whole slide images (WSIs) of diagnostic haematoxylin and eosin (H&E) slides from the BRAF, MISSONI, and VALENTINO cohorts were acquired using a Zeiss AxioScan.Z1 slide scanner (Carl Zeiss AG, Oberkochen, BW, Germany) (40× magnification, 0.11 μm/pixel). Colorectal liver metastasis tissue samples (EPICC) were obtained from Cancer Tissue Bank (https://www.cancertissuebank.org/) for analysis. WSIs from the EPICC cohort, which composed part of the training dataset for the cell classifier, were scanned using a Hamamatsu NanoZoomer slide scanner (Hamamatsu Photonics, Hamamatsu, Shizuoka, Japan) (40× magnification, 0.22 μm/pixel). For interoperability of the images originating from different scanners, image resizing and sharpening steps were applied to ensure that the images were of a consistent resolution and appearance.

### Multiplex immunofluorescence

The multiplex immunofluorescence (mIF) images were acquired using the Co‐Detection by Indexing (CODEX) platform (Akoya Biosciences, Marlborough, MA, USA), imaged with a Keyence microscope (Keyence Corp., Osaka, Japan) (0.5 μm/pixel). The mIF panel consisted of 17 antibodies (supplementary material, Table S2). Those used to detect CD4, CD8, CD20, CD3e, CD68, CD31, Ki67, pan‐cytokeratin, and CD11c were inventory antibodies, fluorescence‐conjugated by Akoya Biosciences. FSP1, αSMA, vimentin, CD163, FOXP3, PMS2, MSH6, and LGR5 were purified commercial antibodies, manually conjugated following Akoya's instructions.

Following acquisition of the mIF images, the same section was stained with H&E to enable direct comparison between tissue morphology and mIF marker expression. To allow the H&E section to be imaged correctly, the coverslip bearing the tissue section was mounted onto a standard microscope slide. Images of the H&E‐stained slides were acquired using a Zeiss AxioScan.Z1 slide scanner (40× magnification, 0.11 μm/pixel). For the interoperability of the H&E and mIF images, the H&E images were subsequently resized and flipped to match the resolution and orientation of the mIF images.

### Image analysis

The cell classification pipeline was composed of two separate components, each governed by a convolutional neural network: a cell detector and a cell classifier.

#### Cell detection

The cell detector used a spatially constrained convolutional neural network (SCCNN) [18] style architecture to identify cell nuclei. As input, the detector received a 31 × 31 pixel tissue image patch (0.44 μm/pixel resolution) and output an 11 × 11 pixel probability map, indicating the probability that a cell nucleus existed at a particular pixel. The cell detector was applied to overlapping patches of the WSI in a sliding window fashion; patches were then arranged into a raw nucleus detection map. The raw detection map was converted into a set of detected cells using a maximum clique method to detect peaks of high pixel intensity. Each detected cell was represented as an (*x*, *y*) coordinate point. The set of detected cells was provided as input to the cell classifier.

#### Cell classification

The cell classifier used a DenseNet‐201 architecture [30] to assign a cell label to each detected cell. To improve classification accuracy, the convolutional layers of the network were pre‐trained on the ImageNet dataset before being re‐trained on the problem of cell classification. The classifier can identify eight different cell types commonly found in colorectal tissue: cancer epithelial cells, normal epithelial cells, fibroblasts, lymphocytes, neutrophils, macrophages, endothelial cells, and myocytes. In addition, the classifier has a ninth class, unknown, which is assigned to cells and other objects that the classifier is unable to identify. For each detected cell, we collected a 51 × 51 pixel image patch (0.44 μm/pixel resolution), centred at the cell's detected position. After being resized to 224 × 224 pixels, these image patches were sent to the classification model, and for each patch the model output the probabilities for the nine cell classes. Each cell was classified as the type with the highest probability. However, if this probability was below the minimum threshold value of 50%, the cell was reassigned to ‘unknown’. Each classified cell was represented by its detected (*x*, *y*) position and its classified type. These cell data triplets formed the basis of our spatial cell analysis.

#### Training data collection

Training data were generated through manual annotation of WSIs by an experienced gastrointestinal pathologist (MF). Each annotation was a single coordinate point, located at the cell's nucleus and colour coded according to its type (supplementary material, Figure S2). Annotations were collected for eight distinct cell types, along with additional annotations of non‐cellular objects and possible artefacts, which were assigned to the ninth ‘unknown’ class. Annotations were predominantly collected from the EPICC cohort, with some additional annotations from the BRAF and MISSONI cohorts. In total, 38,321 cells were annotated within the cohort of 33 metastatic CRC (mCRC) WSIs from 23 patients.

### Spatial analysis

For each cell of interest, its neighbourhood was defined as a circle with a radius of 100 pixels (equivalent to 44 μm), centred at its detected position. A cell was considered to be tumour‐associated if there was a cancer epithelial cell present within this neighbourhood. In this work, we focused on three subgroups of tumour‐associated cells: lymphocytes, macrophages, and endothelial cells. For each slide, we recorded the count of cancer epithelial cells and the counts of each type of tumour‐associated cell. Slide‐level metrics were aggregated into patient‐level metrics by taking the mean across all samples belonging to the patient.

### Survival analysis

Each cell metric was tested in a univariate setting using a two‐sided log‐rank test, with PFS as the chosen endpoint. For lymphocytes and macrophages, tumour‐associated cell abundance was measured directly from the patient‐level cell counts. For endothelial cells, tumour‐associated cell abundance was measured as the ratio between the patient‐level intra‐tumour endothelial cell count and the patient‐level cancer cell count. Metrics that were significant in the univariate setting (*p* ≤ 0.05) were also tested in a multivariate Cox proportional hazards model. Due to differences in the available clinical data, metrics significant in the VALENTINO cohort were tested in a separate model. For the BRAF and MISSONI cohort model, the following clinical variables were included: age, tumour grade, treatment, and microsatellite status. For the VALENTINO cohort model, the following clinical variables were included: age and trial arm.

#### Validation of cell classifier with multiplex immunofluorescence

Cell segmentation and marker quantification of the mIF image data were performed using the CODEX Processor software. Detected cells were subsequently grouped into cell types by manual gating of the markers specific to the given type. The corresponding H&E WSI was analysed with the cell classification pipeline to produce a matching set of cells. An automated registration pipeline [31] was then applied to align the two sets of detected cells. The tissue section was broken down into windows of 1,887 × 1,887 μm, or 5,000 × 5,000 pixels relative to the mIF image data. Windows manually identified as containing a significant proportion of autofluorescence were discarded. In the remaining windows, the counts of cells detected, in both mIF and H&E, were recorded for each cell type. Spearman correlation was used to measure the concordance of the counts. The confidence intervals (CIs) for the scatter plot were computed by fitting a linear model.

For further information, please refer to Supplementary materials and methods.

## Results

### Single‐cell classification with DL in metastatic CRC

We trained a single‐cell classifier to identify cell nuclei on standard H&E sections and categorised them into eight different cell types (Figure 1C). By studying the cellular balance on a local level, spatial maps of cancer, mesenchymal stromal, and immune cells were generated (Figure 1D), providing a window into the ecological balance of tumours. Those maps encoded not only the density of tumour cells (red), lymphocytes (green), and fibroblasts (blue), for example, but also highlighted areas where tumour cells were in close proximity to lymphocytes (yellow) or fibroblasts (magenta), as in the example shown in Figure 1D.

### Validation with multiplex immunofluorescence

**Figure 2:**
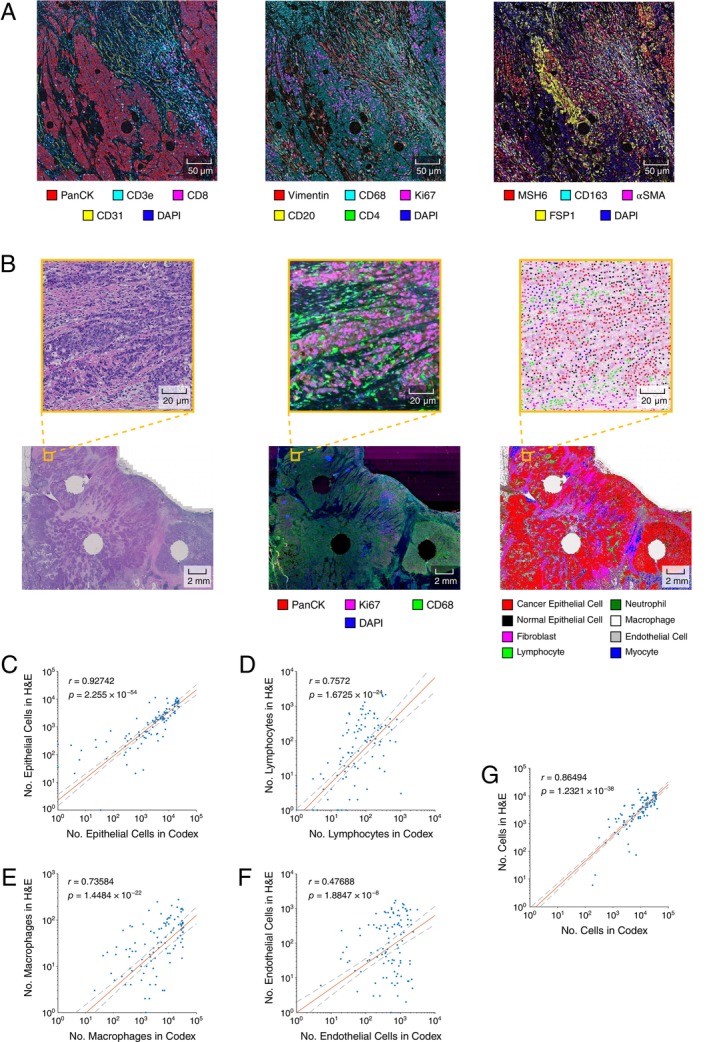
Multiplex immunofluorescence validation. (A) Demonstration of highly mIF from CODEX platform on a selection of 14 markers. (B) Aligned H&E (left) and CODEX (centre) images. Images are aligned using an automated registration algorithm. With the application of the cell classifier to the coregistered H&E image, classified cells (right) can be directly associated with mIF marker expression. (C–F) Comparison of numbers of cells detected across the full CODEX cohort, according to H&E and mIF. H&E and mIF images are registered and subdivided into square windows, with counts of detected cells of a given cell type reported for each window. (C) Comparison of detected epithelial cells in H&E and mIF. Epithelial cells in mIF images were identified through manual gating of pan‐cytokeratin marker. (D) Comparison of detected lymphocytes in H&E and mIF. Lymphocytes in CODEX image were identified through manual gating of CD3e, CD4, CD8 and CD20 markers. (E) Comparison of detected macrophages in H&E and mIF. Macrophages in mIF image were identified through manual gating of CD68 marker. (F) Comparison of detected endothelial cells in H&E and mIF. Endothelial cells in mIF image were identified through manual gating of CD31 marker. (G) Comparison of total detected cells in H&E and mIF. Cells in mIF image were identified by CODEX Processor software.

Using the CODEX platform in conjunction with an H&E image of the same histological section (Figure 1E), we validated our manual annotation‐based H&E cell classifier with a 17‐marker mIF panel. This panel included markers specific to the key cell types identified in our H&E analysis: lymphocytes, macrophages, and endothelial cells. This panel also allowed us to further subclassify these cell types. For lymphocytes, the panel contained markers for B cells (CD20), T cells (CD3e), cytotoxic T cells (CD8), helper T cells (CD4), and regulatory T cells (FOXP3). For macrophages, the panel contained markers for M1 (CD11c) and M2 (CD163) phenotypes, as well as a marker expressed by both (CD68) (Figure 2A).

We applied the mIF panel to 14 slides from the MISSONI cohort and eight slides from the BRAF cohort. By detecting the presence of the mIF markers associated with each cell type, we classified cells in the mIF with labels corresponding to those used by the H&E classifier. This provided an orthogonal set of cell labels for the same section, which, following automated alignment of the H&E and mIF samples (Figure 2B), were used as a reference set to validate the output of the cell classifier. We evaluated the concordance of our H&E and mIF‐derived cell labels by dividing the aligned images into square windows and comparing the counts of cells within those windows (Figure 2C–G). The correlations between our classifier and mIF were confirmed to be significant for epithelial cells (Figure 2C), lymphocytes (Figure 2D), macrophages (Figure 2E), endothelial cells (Figure 2F), and total cells (Figure 2G).

### Colocalisation of cancer, stroma, and immune cells

**Figure 3:**
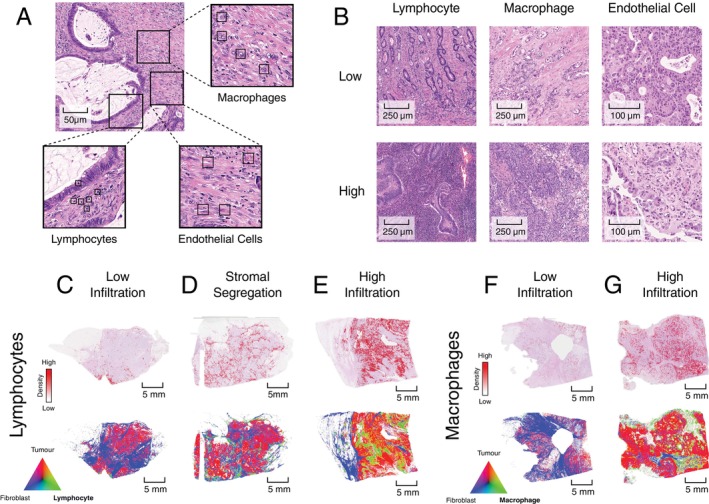
Transforming a standard H&E slide into a density and colocalisation map of cancer, mesenchymal stromal, and immune cells. (A) Examples of cell types of interest. (B) High‐ and low‐density regions for stromal cell types of interest. (C–E) Variation in lymphocyte distribution. Top: density of tumour colocalising lymphocytes (red = high, white = low); bottom: regional balance of cell types (green = lymphocytes, red = tumour cells, blue = fibroblasts). (C) Low tumour infiltration. (D) Lymphocytes segregated from tumour by stroma. (E) High tumour infiltration. (F, G) Variation in macrophage distribution. Top: density of tumour colocalising macrophages (red = high, white = low); bottom: regional balance of cell types (green = macrophages, red = tumour cells, blue = fibroblasts). (F) Low tumour infiltration. (G) High tumour infiltration.

Leveraging the output of the automatic whole slide cell classification pipeline, we can identify regions rich in a particular cell type, notably lymphocytes, macrophages, and endothelial cells (Figure 3A). Densities of these cell types can vary substantially between and within samples (Figure 3B). Thus, using the raw cell classification data, we generated cell density maps, showing the balance of three selected cell types. For each map, the slide was divided into regions of size 88 × 88 μm, each corresponding to a pixel in the map and coloured according to the balance of cells in that region (triangular legend, Figure 3C–G). With these maps we were able to identify a variety of cellular configurations across our cohorts. These included regions with very few lymphocytes (Figure 3C) and those with high lymphocytic density (Figure 3D,E). Within those high‐density sections, we observed instances where there was clear segregation between the lymphocytes and the tumour (Figure 3D), as well as others where the lymphocytes and tumour cells were highly intermixed (Figure 3E). High‐ and low‐density regions can also be observed for tumour‐associated macrophages (Figure 3F,G).

### Immune and endothelial cell distribution predicts survival across cohorts

**Figure 4:**
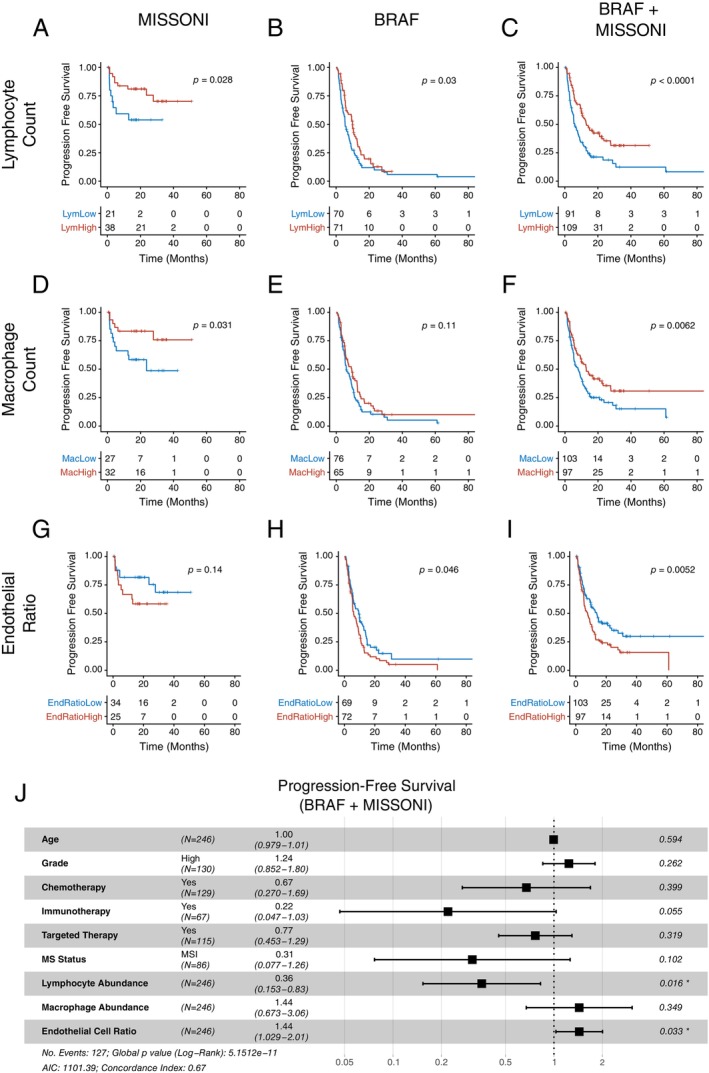
Prognostic value of cellular colocalisation metrics. (A–C) PFS for high/low count of tumour colocalising lymphocytes in MISSONI (A), BRAF (B), and both cohorts (C). (D, F) PFS for high/low count of tumour colocalising macrophages in MISSONI (D), BRAF (E), and both cohorts (F). (G–I) PFS for high/low ratio of tumour colocalising endothelial cells in MISSONI (G), BRAF (H), and both cohorts (I). (J) Cox proportional hazard model of progression‐free survival in BRAF and MISSONI cohorts, using clinical covariates and cell colocalisation metrics.

We first investigated whether the count of colocalised cell types correlated with PFS in the MISSONI and BRAF cohorts. Cells were considered to be colocalised with the tumour if they were within a 44‐μm radius of cancer epithelial cells. We found that lymphocyte infiltration was indeed prognostic in both cohorts independently (Figure 4A,B) and combined (Figure 4C), consistent with previous findings [4, 5, 6, 28]. Macrophage density was prognostic in MISSONI (Figure 4D) but was not significant in BRAF (Figure 4E), although when putting together the two cohorts the Kaplan–Meier curves separated significantly (Figure 4F). Interestingly, intra‐tumour endothelial cell count, although showing only a trend towards a worse prognosis in MISSONI (Figure 4G), was prognostic in the BRAF cohort (Figure 4H) and highly significant when the two cohorts were combined (Figure 4I).

We found that lymphocyte density was indeed robust to multivariate analysis (Figure 4J, HR = 0.36, 95% CI: 0.153–0.83), indicating a more favourable prognosis. Macrophage count was not, possibly because we could not separate M1 from M2 macrophage phenotypes, which are divergent prognostic indicators [12, 32, 33, 34]. Importantly, endothelial cell density was significant in the multivariate analysis (Figure 4J, HR = 1.44, 95% CI: 1.029–2.01).

**Figure 5:**
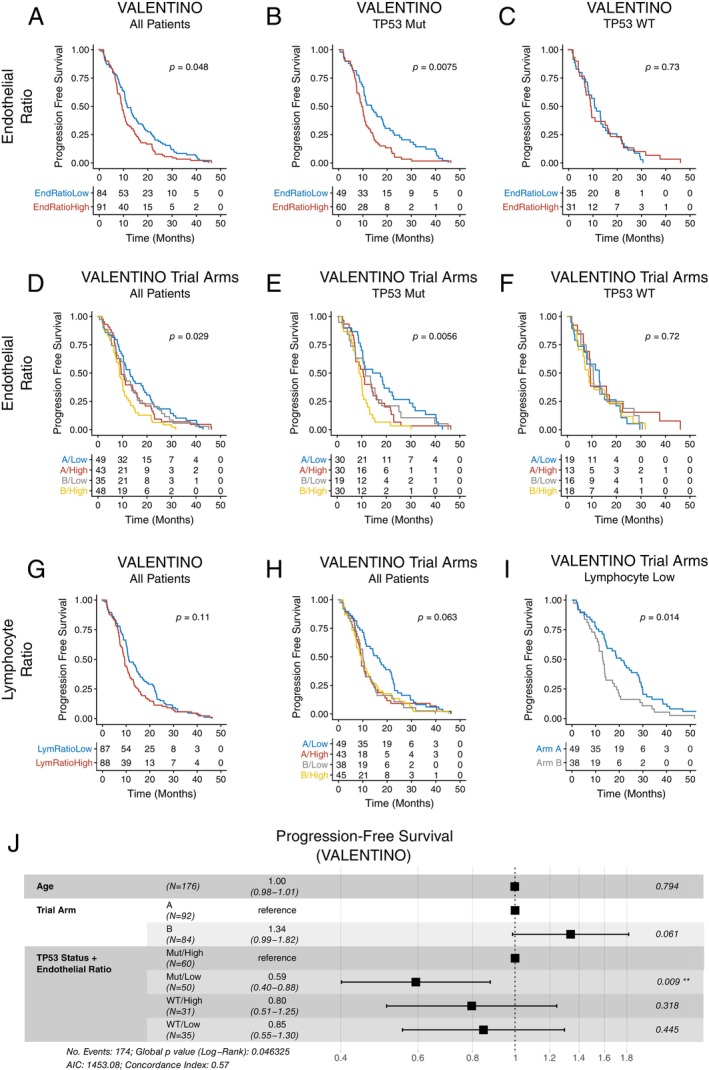
(A–F) Prognostic value of cellular colocalisation metrics in VALENTINO cohort. (A–F) PFS for high/low ratio of tumour colocalising endothelial cells. (A, D) All patients, (B, E) *TP53* mutant patients, (C, F) *TP53* WT patients. Tables below indicate numbers at risk. (G, H) PFS for high/low ratio of tumour colocalising lymphocytes. (I) Comparison of PFS by trial arm in lymphocyte low patients. (J) Cox proportional hazard (CPH) model of PFS in VALENTINO cohorts, using clinical covariates and combined *TP53* status and tumour endothelial cell ratio.

Intrigued by the results on endothelial cells in the MISSONI and BRAF cohorts, we applied the same measurement to the VALENTINO cohort and indeed found that endothelial cells were a significant predictor of PFS (Figure 5A) and in a *TP53*‐specific manner (Figure 5B,C). Following separation by trial arm, the significance of endothelial cell density as a predictor of PFS was retained (Figure 5D) and indeed was *TP53*‐dependent (Figure 5E,F). This combination of *TP53* status and level of endothelial–tumour cell colocalisation was also found to be significant in the multivariate analysis (Figure 5J).

In contrast to the findings in the BRAF and MISSONI cohorts, we also found that elevated lymphocyte ratio was associated with poorer PFS in VALENTINO (Figure 5G), although this trend was not significant. However, when we stratified VALENTINO patients by trial arm, we observed a clear difference in PFS for lymphocyte‐low patients compared to lymphocyte‐high patients (Figure 5H). For lymphocyte‐low patients, those in arm A had significantly better PFS (Figure 5I), whereas in lymphocyte‐high patients, both trial arms exhibited the same pattern of poor survival, comparable to lymphocyte‐low patients in arm B. This may suggest that only patients with immune‐cold tumours benefitted from the addition of maintenance chemotherapy.

To evaluate the effect of the 44‐μm radius used to define cells as tumour infiltrating, we recalculated our metrics with two other radii: 22 and 88 μm. While this impacted the raw numbers of TILs, macrophages, and endothelial cells identified, their counts nevertheless remained very highly correlated with the counts made using the 44‐μm radius (supplementary material, Figure S3A–F). In addition, within the BRAF and MISSONI cohorts, we observed the same trend of significantly shorter PFS in patients with high lymphocyte infiltration for all three radii (supplementary material, Figure S4A–C). These findings suggest that our observations are not especially sensitive to the specific radius used for identifying tumour‐infiltrating cells.

## Discussion

CRC is a disease exhibiting a diverse histological presentation. While some CRC subtypes, such as MSI/MSS, are known to have substantially different survival rates, there are still considerable differences in survival and therapeutic efficacy across patients of the same subtype. Thus, further stratification is needed to better characterise this disease. Computational histopathology algorithms offer an objective and data‐driven means to explore this problem, allowing for the characterisation of spatial cellular patterns that are challenging to quantitatively assess through visual inspection. However, it is key that these computational metrics be designed, applied, and validated in well‐characterised trial cohorts with extensive clinical information. In this work, we demonstrated that cellular metrics of tumour infiltration, quantified computationally using AI, are prognostic in terms of PFS within our clinical trial cohorts.

Previous studies identified the prognostic roles of TILs [5, 35, 36] and tumour‐associated macrophages [12, 37, 38]. By leveraging our automated cell classification pipeline, we have been able to show that an increase in infiltration of both lymphocytes and macrophages are significantly associated with improved PFS. However, further analysis is needed to understand the specific biological mechanisms underpinning these relationships. It must be stressed that the immune cell types identified by the AI classifier are broad categorisations that encompass a range of cellular lineages. For instance, macrophages are commonly divided into the M1 and M2 subtypes, which are believed to be associated with pro‐ and anti‐inflammatory responses respectively. These subtypes are difficult to distinguish within a standard H&E section, which limits the capability of purely H&E‐based image analysis. While it is challenging to perform at a comparable scale, this type of cellular subtyping can be achieved with mIF, such as CODEX, which will form future work. In addition, our H&E analysis used proximity of immune and tumour cells as an indicator of a potential cell–cell interaction. However, it is not possible to confirm the existence of any cellular interaction from proximity alone. Thus, it will be important to quantify interactions between the cell types of interest more directly, using modern spatial transcriptomic platforms, for instance.

In this study, we focused on specific, clinically annotated CRC cohorts and identified for the first time endothelial cell populations as independently prognostic across patient cohorts. Perhaps the most likely explanation for the finding of increased risk of progression in patients with an elevated abundance of endothelial cells, proximal to cancer epithelial cells, is that this metric acts as a surrogate for vascular invasion, a histopathological feature known to be associated with poor outcome in patients with CRC [39, 40].

Importantly, we combined our H&E‐based AI classifiers with novel highly mIF assays, through which the cell types identified on H&E stained slides can be further divided into functionally distinct subtypes. Through our validation work, we have shown that the cell types identified in H&E images may be further investigated using mIF platforms. Unfortunately, this approach is unlikely to be feasible at scale due to the significantly higher costs and time associated with generating mIF data. However, if this modality is combined with H&E, there is an opportunity to use mIF imaging as a reference to automatically generate cell annotations in H&E and, consequently, generate training datasets at scales beyond what experienced pathologists could manually curate. Furthermore, this might also allow us to train AI models to identify mIF‐derived cell subtypes directly on H&E slides. Previous works [41, 42, 43] showed that AI models might be able to identify MSI status, a feature of colorectal cancer commonly identified through immunohistochemistry, using only H&E WSIs, which illustrates the potential of such an approach.

Endothelial cells are particularly difficult to identify and classify due to their considerable morphological variation, which may make generating a comprehensive training dataset challenging for this cell type. Given that we see a lower correlation between detected endothelial cells in H&E and CD31+ cells detected using mIF, it is possible that the classifier, which was trained on cells manually identified in H&E, may not be capturing the full variation of endothelial cells present in CRC tissue. We also observed instances of under‐detection of CD31+ cells by the CODEX processor software, perhaps for similar reasons, which may also contribute to the lower correlation. Further investigation will be required to understand the biological implications of this limitation of the classification, as regards the associations between tumour‐infiltrating endothelial cell abundance and patient outcome that we have identified. However, this also illustrates another potential future benefit of mIF‐guided training of H&E‐based AI models, by providing a more direct identification of cell types that are sometimes visually difficult to discern on H&E.

Ultimately, features and metrics identified using computational pathology might aid progress towards a more comprehensive description of tumour biology and, in combination with clinically annotated cohorts, identify important patient subsets whose response to treatment differs and, thus, may benefit from escalation or de‐escalation of their treatment. Further, these quantitative descriptions of TME ecology can also serve as an important starting point to a deeper understanding of the underlying mechanisms governing the TME, which may ultimately lead the way to novel treatments.

These instances of personalised medicine, derived through computational histopathology, are beyond the scope of this work and require rigorous validation on large independent cohorts. However, several computational pathology‐derived biomarkers have already been clinically validated and received regulatory approval [44, 45], which demonstrates the pathway for further developments of this kind. Of note is the DoMore clinical decision support model [27], a DL‐based CRC risk predictor that has been validated on clinical cohorts exceeding 1,000 patients. While the process of clinical implementation does present many additional considerations and will ultimately require biomarkers to be tested within prospective clinical trials, we believe that an AI‐driven investigation of the TME, evaluated within well‐annotated clinical trial data, provides a strong basis for identifying the prognostic characteristics of tumour biology that can subsequently be tested within the clinic.

## Author contributions statement

NT designed and trained the cell classifier, designed cell‐based metrics and interpreted the results. CS generated multiplex immunohistochemistry data and interpreted the results. AB generated the training data and assisted in training for the cell classifier. SHL generated the training data and assisted in training for the cell classifier. AMB assisted in the generation of multiplex immunohistochemistry data. HMK contributed to clinical data analysis and interpretation. VA, FM, FB, GM, RI, CC, FP and SL helped with sample preparation and data generation and also contributed to clinical data analysis and interpretation. GC helped with data analysis. TG assisted with data generation and data analysis. MF generated the training data, assisted in training the cell classifier and contributed to sample generation. AS contributed to data generation and data analysis. SL and MF collected the clinical cohorts. SL, MF and AS supervised the study. NT, MF and AS wrote the manuscript. All other authors contributed to writing the manuscript.

## Supporting information


Supplementary materials and methods
**Figure S1.** Stacked bar charts comparing distribution of patients in BRAF, MISSONI, and VALENTINO cohorts for selected clinical variables
**Figure S2.** Example illustration of annotated cells from training dataset
**Figure S3.** Comparisons of numbers of tumour‐infiltrating cells using different maximum distances (BRAF and MISSONI cohorts, one point per slide)
**Figure S4.** Kaplan–Meier (KM) plots showing associations between infiltrating lymphocytes and progression‐free survival for combined BRAF and MISSONI cohorts using different radii for detecting infiltrating cells
**Table S1.** Clinical data for the three cohorts
**Table S2.** Optimised panel of antibodies used for multiplex immunohistochemistry

## Data Availability

Code is available as a GitHub list: https://github.com/stars/ntrahearn/lists/crc-classifier. Analysed data are available at Mendeley: https://data.mendeley.com/datasets/9z48yfzr63. Digital WSIs of H&E‐stained tissue sections are available at BioStudies: https://www.ebi.ac.uk/biostudies/bioimages/studies/S-BIAD1407. The highly mIF images are available upon reasonable request.
