# Supplementary material for: Computational pathology applied to clinical colorectal cancer cohorts identifies immune and endothelial cell spatial patterns predictive of outcome
Source: J Pathol. 2025 Jan 9;265(2):198–210. doi: 10.1002/path.6378 (PMC11717494; doi:10.1002/path.6378)
Supplement: Supplementary file 1 — Supplementary materials and methods Figure S1. Stacked bar charts comparing distribution of patients in BRAF, MISSONI, and VALENTINO cohorts for selected clinical variables Figure S2. Example illustration of annotated cells from training dataset Figure S3. Comparisons of numbers of tumour‐infiltrating cells using different maximum distances (BRAF and MISSONI cohorts, one point per slide) Figure S4. Kaplan–Meier (KM) plots showing associations between infiltrating lymphocytes and progression‐free survival for combined BRAF and MISSONI cohorts using different radii for detecting infiltrating cells Table S1. Clinical data for the three cohorts Table S2. Optimised panel of antibodies used for multiplex immunohistochemistry [file PATH-265-198-s001.zip › path6378-sup-0001-SuppMatMeth,TablesS1,S2.docx]

**Computational pathology applied to clinical colorectal cancer cohorts identifies immune and endothelial cell spatial patterns predictive of outcome**

N Trahearn *et al. J Pathol* <https://doi.org/10.1002/path.6378>

# **Supplementary materials and methods**

**Supplementary Table S1 is provided as a separate Excel file**

**Supplementary Table S2**

# **Supplementary materials and methods**

# Ethical approval and patient consent

Ethical approval was given by local ethics committees (BRAF: Oncologic Institute of Veneto, 2017/34. Missoni: Fondazione IRCCS Istituto Nazionale dei Tumori di Milano Institutional Review Board, INT 117/15. Valentino: Fondazione IRCCS Istituto Nazionale dei Tumori di Milano Institutional Review Board, INT 70/15, EPICC: 2015/2/QM/TG/CaCOL). Consent was given by all patients involved in the study. All samples were handled in compliance with the Declaration of Helsinki. Clinical and sample data (supplementary material, Table S1) were managed using anonymous numerical codes. Research was performed in accordance with local and national ethical standards.

# Data acquisition and pre-processing

Digital whole-slide images (WSIs) of diagnostic H&E slides from the BRAF, MISSONI, and VALENTINO cohorts were acquired using a Zeiss AxioScan.Z1 slide scanner (Carl Zeiss AG, Oberkochen, BW, Germany) (40× magnification, 0.11 µm/pixel). Colorectal liver metastasis tissue samples (EPICC) were obtained from Cancer Tissue Bank (<https://www.cancertissuebank.org/>) for analysis. WSIs from the EPICC cohort, which composed part of the training dataset for the cell classifier, were scanned using a Hamamatsu NanoZoomer (Hamamatsu Photonics, Hamamatsu, Shizuoka, Japan) scanner (40× magnification, 0.22 µm/pixel).

For interoperability of the images originating from different scanners, additional pre-processing steps were applied to ensure that the images were of a similar appearance and format. Slide images were converted to a set of JPEG tiles of size 2,000 × 2,000 pixels, at a resolution of 0.44 µm/pixel, spanning the entire image with no overlap. To increase the visual similarity of images originating from different scanners, tiles taken from AxioScan images were also digitally sharpened with unsharp masking, with radius = 5 and amount = 2.

Multiplex Immunofluorescence

The multiplex immunofluorescence (mIF) images were acquired using the Co-Detection by Indexing (CODEX) platform (Akoya Biosciences, Marlborough, MA, USA), imaged with a Keyence 800 microscope (Keyence Corp., Osaka, Japan) (0.5 μm/pixel). CODEX Instrument Manager (version 1.30.0.12) was used to acquire mIF images. The mIF panel consisted of 17 antibodies (supplementary material, Table S2). Of those, CD4, CD8, CD20, CD3e, CD68, CD31, Ki67, PCK, and CD11c were inventory antibodies, conjugated by Akoya Biosciences. The remaining antibodies, FSP1, αSMA, Vimentin, CD163, FOXP3, PMS2, MSH6, and LGR5, were purified commercial antibodies free of carrier proteins; these antibodies were manually conjugated as per Akoya’s instructions. Due to the specific requirements of the CODEX fluidics system, the formalin-fixed paraffin-embedded (FFPE) tissue sections used for mIF were mounted directly onto poly-L-lysine coated coverslips (22 ×22 mm) following Akoya’s instructions (<https://akoyabio.com/wp-content/uploads/2021/01/CODEX-User-Manual.pdf>). Staining and imaging for the mIF samples were performed on the coverslip-mounted section. Subsequent to the acquisition of the mIF image, cells were detected automatically by the CODEX Processor software (version 1.7.0.6)

Following the acquisition of the mIF images, the same section was subsequently stained with H&E to enable direct comparison between tissue morphology and mIF marker expression. To allow the H&E-stained section to be imaged correctly, the coverslip was then mounted onto a standard superfrost microscope slide using Pertex mounting medium. Images of the H&E-stained slides were acquired with the Zeiss AxioScan.Z1 slide scanner (40× magnification, 0.11 µm/pixel). For the interoperability of the H&E and mIF images, the H&E images were subsequently resized to match the resolution of the mIF images. It should be noted that, due to differences in their imaging systems, the Keyence and Zeiss scanners image opposite sides of the tissue section. Thus, the H&E image was also flipped horizontally to account for this difference.

# Image analysis

The cell classification pipeline was composed of two separate components, each governed by a convolutional neural network: a cell detector and a cell classifier.

## Cell detection

The cell detector uses a spatially constrained convolutional neural network (SCCNN)^18^ style architecture to identify cell nuclei. The detection pipeline was implemented in Python using the TensorFlow library.

As input, the detector receives a 31×31 pixel tissue image patch (0.44 μm/pixel resolution) and outputs an 11×11 pixel probability map, indicating the probability that a cell nucleus exists at a particular pixel. To perform detection on a WSI, the cell detector is run on patches of the image in a sliding window fashion, which are then composed into a raw nucleus detection map. Locations where cell nuclei are likely to exist will appear as peaks of high pixel intensity in the raw detection map. The raw detection map is converted into a set of detected cells using a maximum clique method to detect the peaks. Each detected cell is represented as an (*x*, *y*) coordinate point. The set of detected cells is provided as input to the cell classifier.

## Cell classification

The cell classifier uses a DenseNet-201 architecture [30] to assign a cell label to each detected cell. To improve classification accuracy, the convolutional layers of the network were pre-trained on the ImageNet dataset before being re-trained on the problem of cell classification. The cell classifier was implemented in Python using the PyTorch library.

The classifier can identify eight different cell types commonly found in colorectal tissue: cancer epithelial cells, normal epithelial cells, fibroblasts, lymphocytes, neutrophils, macrophages, endothelial cells, and myocytes. In addition, the classifier has a ninth class, unknown, which is assigned to cells and other objects that the classifier is unable to identify. As input, the classifier receives a 51×51 pixel image patch (0.44 μm/pixel resolution), centred at the cell’s detected position. Due to the pre-trained model’s architecture, the image patch is subsequently resized to 224×224 pixels to fit the required input size. From this patch the classifier outputs a probability vector of length 9, each value indicating the classifier’s certainty that the image patch is an instance of the associated cell type.

Thus, for each detected cell, we take a 51×51 pixel image patch centred at the cell’s detected position. After being resized to 224×224, these image patches are provided to the classification model, and for each cell, the cell class with the highest probability is chosen as the cell type. However, to ensure that only cells that the classifier has identified with high certainty are used in our analysis, cells where the highest probability is below the minimum threshold value of 50% were reassigned to ‘unknown’. Each classified cell is represented by its detected (*x*, *y*) position and its classified type. These cell data triplets form the basis of our spatial cell analysis.

## Training data collection

Training data were generated through manual annotation of WSIs by an experienced gastrointestinal pathologist (MF). Each annotation was single point, located at the cell’s nucleus and colour coded according to its type (supplementary material, Figure S2). Annotations were collected for eight distinct cell types, along with additional annotations of non-cellular objects and possible artefacts, which were assigned to the ninth ‘unknown’ class.

For each annotated cell, the (*x*, *y*) position of the cell and its associated cell type were recorded. The same set of annotated cells was used to train both the cell detection and cell classification models. Thus, these raw annotation data were subsequently converted into image patches and labels of an appropriate format for the associated model.

Cell annotations were predominantly collected from WSIs within the EPICC cohort. However, additional annotations from the BRAF and MISSONI cohorts were included to improve cell identification for images originating from the AxioScan slide scanner. In total, 38,321 cells were annotated within the cohort of 33 metastatic colorectal cancer WSIs from 23 patients.

# Heatmaps of cellular balance

For cellular balance heatmaps, the slide was divided into regions of size 88×88 μm, with a 77-μm overlap between adjacent regions. Within the region we recorded the counts of the three chosen cell types, which were converted into percentages of the total cells in the region. The percentages were used as trilinear coordinates and projected onto a Maxwell Colour Triangle to derive the RGB colour of the pixel corresponding to the region. To prevent the over-representation of regions containing a small number of cells, regions with fewer than 10 cells belonging to the three chosen types had their RGB values scaled by the number of cells divided by 10. The resultant pixels were assembled spatially to produce the final colour heatmap.

# Spatial analysis

For spatial analysis, we wished to identify cells that were tumour-adjacent and, thus, more likely to be interacting with the tumour in a way that may influence its progression. For each cell of interest, we considered its neighbourhood to be a circle, centred at its detected position, of radius 100 pixels (equivalent to 44 μm). From this definition, we defined a cell to be tumour-associated if there was a cancer epithelial cell present within this neighbourhood. In this work we focused on three subgroups of tumour-associated cells: lymphocytes, macrophages, and endothelial cells. For each slide, we recorded the count of cancer epithelial cells and the counts of each type of tumour-associated cell. Slide-level metrics were aggregated into patient level metrics by taking the mean across all samples belonging to the patient. Across the cohorts considered, the mean number of samples per patient was 1.22, with 81% of patients having a single section available for analysis.

# Survival analysis

Each cell metric was tested in a univariate setting using a two-sided log-rank test, with progression-free survival (PFS) as the chosen endpoint. For lymphocytes and macrophages, tumour-associated cell abundance was measured directly from the patient-level cell counts. For endothelial cells, tumour-associated cell abundance was measured as the ratio between the patient-level intra-tumoural endothelial cell count and the patient-level cancer cell count. Metrics that were significant in the univariate setting (*p* ≤ 0.05) were also tested in a multivariate Cox proportional hazards model. Due to differences in the available clinical data, metrics significant in the VALENTINO cohort were tested in a separate model. For the BRAF and MISSONI cohort model the following clinical variables were included: age, tumour grade, treatment type (chemotherapy, targeted therapy, and immunotherapy), and microsatellite status. For the VALENTINO cohort model, the following clinical variables were included: age and trial arm (A or B). All survival analysis was performed in R using the packages survival and survminer.

Validation of cell classifier with mIF

Cell segmentation and marker quantification of the mIF image data, acquired with the AKOYA CODEX platform, was performed using the CODEX Processor software. Detected cells were subsequently characterised into cell types by manual gating of the markers specific to the given type, using the gating functionality within the CODEX MAV software (version 1.5.0.8). The corresponding H&E-stained WSI was analysed with the cell classification pipeline to produce a matching set of cells. The mIF and subsequent H&E images were acquired separately with different machines, so a registration step was required to bring the sets of detected cells into alignment. For this, we applied an automated registration pipeline [31]. Following registration, the image field was broken down into windows of 1,887 × 1,887 µm, or 5,000 × 5,000 pixels relative to the mIF image data. The mIF images were manually inspected for instances of autofluorescence and windows identified as containing a significant proportion of autofluorescence were discarded and not used in for comparison. In the remaining windows the counts of cells detected, in both mIF and H&E, were recorded for each cell type. Spearman correlation was used to measure the concordance of the counts. The confidence intervals for the scatter plot were computed by fitting a linear model. All statistical analysis was performed in R. Scatter plots were generated in R using the ggplot package.

**Table S2.** Optimised panel of antibodies used for multiplex immunohistochemistry.

| **Marker** | **Clone** | **Supplier** | **Catalogue No.** | **Dilution** | **Target** |
| --- | --- | --- | --- | --- | --- |
| CD4 | EPR6855 | Akoya Biosciences, Marlborough, MA, USA | 4550112 | 1 in 200 | T helper cells |
| CD8 | C8/144B | Akoya | 4250012 | 1 in 200 | Cytotoxic T cells |
| CD20 | L26 | Akoya | 4450018 | 1 in 200 | B cells |
| CD68 | KP1 | Akoya | 4550113 | 1 in 100 | Macrophages (M1/M2) |
| CD11c | 118/A5 | Akoya | 4550114 | 1 in 200 | Macrophages (M1) |
| CD163 | EDHu-1 | Bio-Techne, Minneapolis, MN, USA | NB110-48686 | 1 in 200 | Macrophages (M2) |
| CD3e | EP449E | Akoya | 4350020 | 1 in 200 | T cells |
| FOXP3 | 236A/E7 | Thermo Fisher Scientific, Waltham, MA, USA | 14-4777-82 | 1 in 200 | Regulatory T cells |
| CD31 | EP3095 | Akoya | 4450017 | 1 in 100 | Endothelial cells |
| FSP1 | EPR2761(2) | Abcam, Cambridge, UK | ab216003 | 1 in 200 | Fibroblasts |
| Ki-67 | B56 | Akoya | 4250019 | 1 in 50 | Dividing cells |
| PCK | AE-1/AE-3 | Akoya | 4450020 | 1 in 200 | Epithelial cells |
| αSMA | Polyclonal | Abcam | ab5694 | 1 in 200 | Fibroblasts |
| Vimentin | RV202 | Becton, Dickinson, Franklin Lakes, NJ, USA | 550513 | 1 in 200 | Fibroblasts |
| MSH6 | EPR3945 | Abcam | ab214454 | 1 in 50 | DNA repair |
| PMS2 | EPR3947 | Abcam | ab214442 | 1 in 50 | DNA repair |
| LGR5 | GPR49 | Bio-Techne | NBP1-28904 | 1 in 200 | Stem cells |
