# Supplementary material for: Computational pathology applied to clinical colorectal cancer cohorts identifies immune and endothelial cell spatial patterns predictive of outcome
Source: J Pathol. 2025 Jan 9;265(2):198–210. doi: 10.1002/path.6378 (PMC11717494; doi:10.1002/path.6378)
Supplement: Supplementary file 1 — Supplementary materials and methods Figure S1. Stacked bar charts comparing distribution of patients in BRAF, MISSONI, and VALENTINO cohorts for selected clinical variables Figure S2. Example illustration of annotated cells from training dataset Figure S3. Comparisons of numbers of tumour‐infiltrating cells using different maximum distances (BRAF and MISSONI cohorts, one point per slide) Figure S4. Kaplan–Meier (KM) plots showing associations between infiltrating lymphocytes and progression‐free survival for combined BRAF and MISSONI cohorts using different radii for detecting infiltrating cells Table S1. Clinical data for the three cohorts Table S2. Optimised panel of antibodies used for multiplex immunohistochemistry [file PATH-265-198-s001.zip › path6378-sup-0002-FiguresS1-S4.pdf]

Computational pathology applied to clinical colorectal cancer cohorts identifies immune and endothelial cell spatial patterns predictive of outcome

N Trahearn *et al. J Pathol* https://doi.org/10.1002/path.6378

Supplementary Figures S1–S4

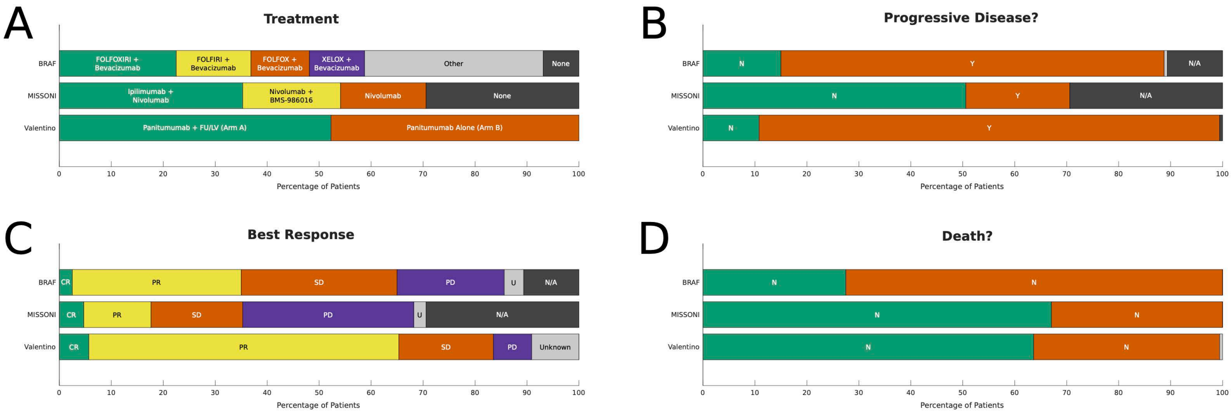

Figure S1. Stacked bar charts comparing distribution of patients in the BRAF, MISSONI, and VALENTINO cohorts for selected clinical variables. (A) Treatment regimen. (B) Progressive Disease. (C) Best Response to Treatment. (D) Death.

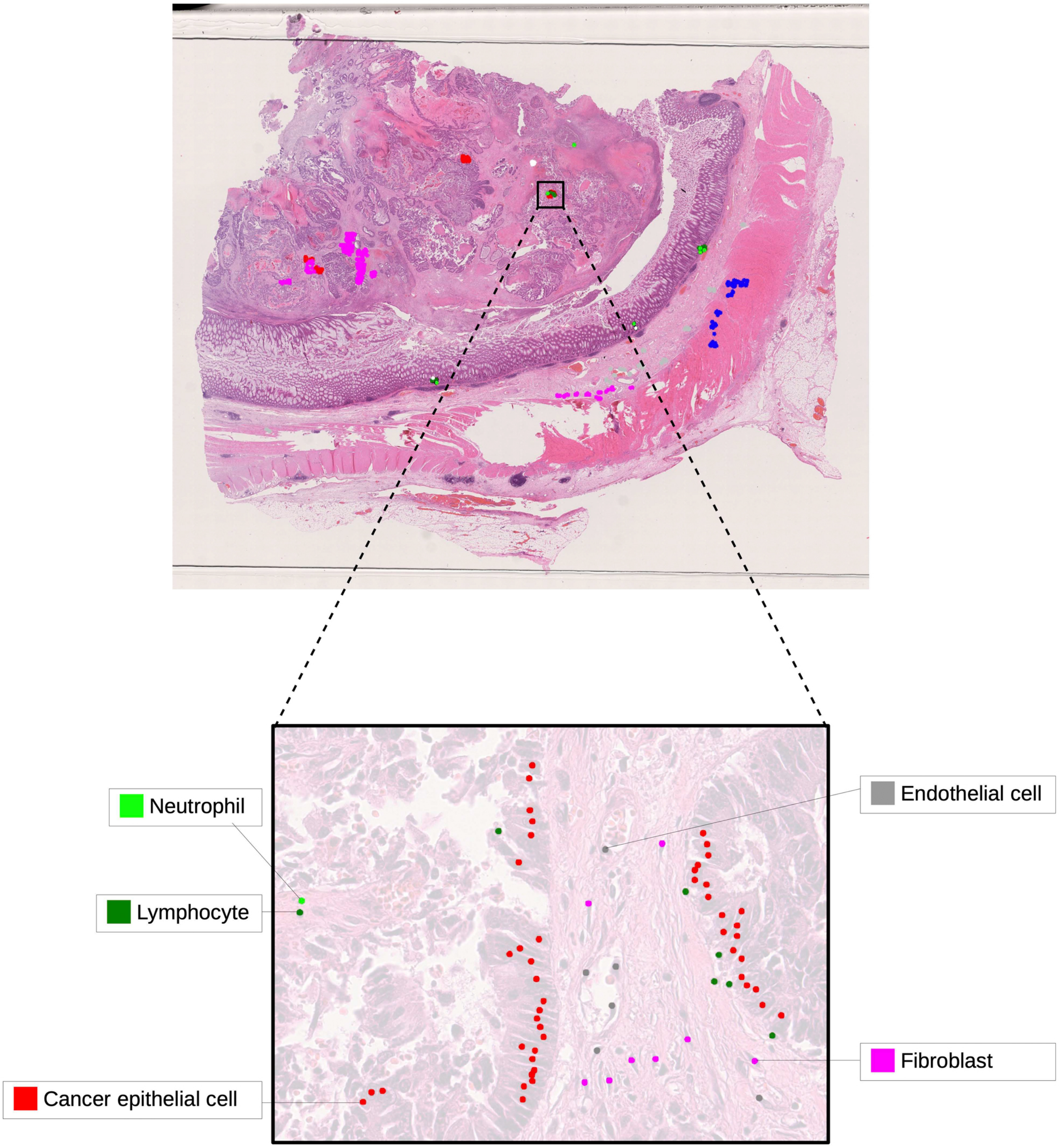

Figure S2. Example illustration of annotated cells from training dataset.

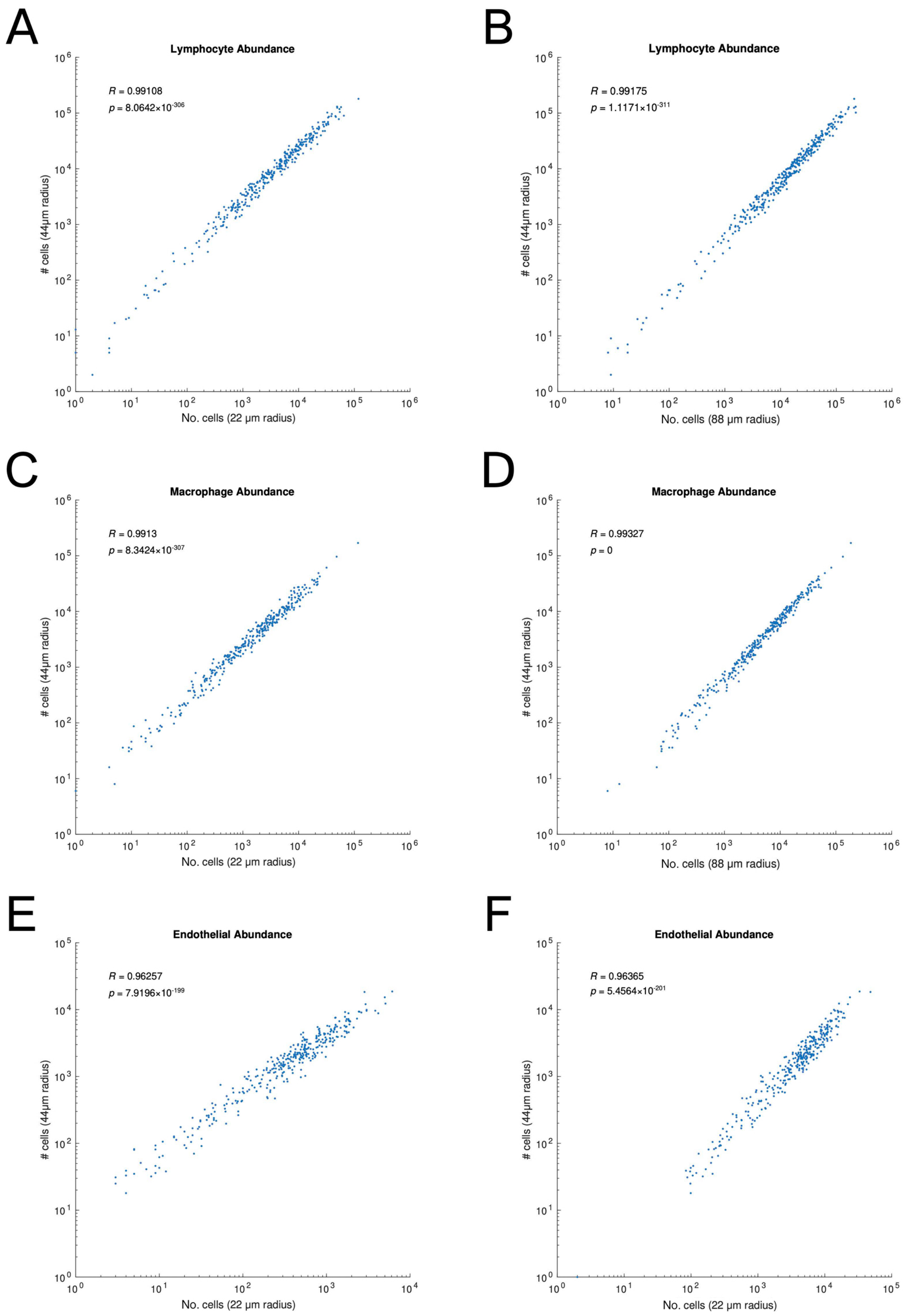

Figure S3. Comparisons of numbers of tumour infiltrating cells, using different maximum distances (BRAF and MISSONI cohorts, one point per slide). (A) Lymphocytes 22 μm versus 44 μm. (B) Lymphocytes 88 μm versus 44 μm. (C) Macrophages 22 μm versus 44 μm. (D) Macrophages 88 μm versus 44 μm. (E) Endothelial cells 22 μm versus 44 μm. (F) Endothelial Cells 88 μm versus 44 μm. High value Spearman correlations are seen for all comparisons (minimum of 0.96).

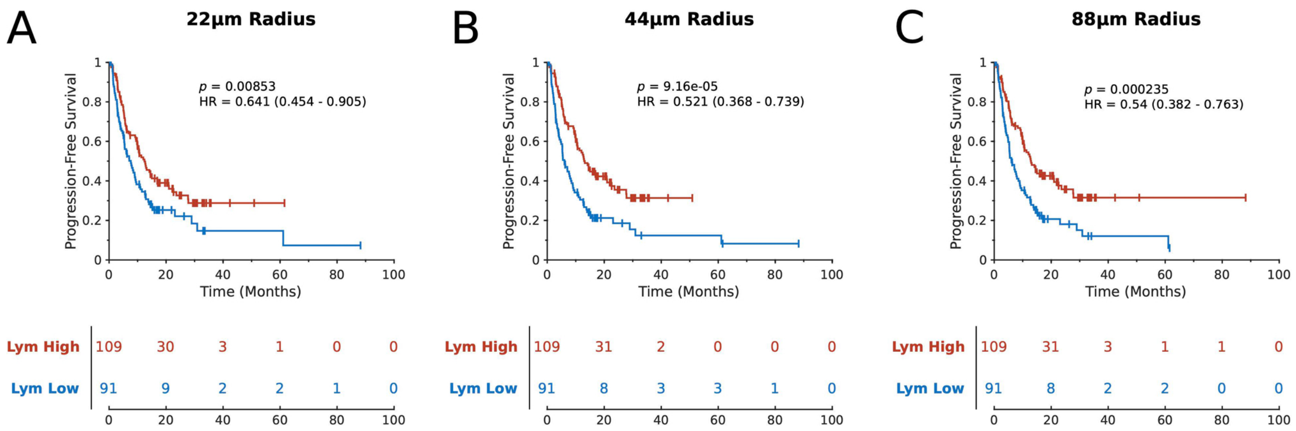

Figure S4. Kaplan–Meier (KM) plots showing the associations between infiltrating lymphocytes and progression-free survival for the combined BRAF and MISSONI cohorts, using different radii for detecting infiltrating cells. (A) 22 µm radius. (B) 44 µm radius. (C) 88 µm radius. All three radii produced significant associations with PFS, as calculated by a log-rank test.
